# The Effect of Temperature on the Hypersensitive Response (HR) in the *Brassica napus–Leptosphaeria maculans* Pathosystem

**DOI:** 10.3390/plants10050843

**Published:** 2021-04-22

**Authors:** Cunchun Yang, Zhongwei Zou, Wannakuwattewaduge Gerard Dilantha Fernando

**Affiliations:** Department of Plant Science, Faculty of Agriculture and Food Science, University of Manitoba, Winnipeg, MB R3T 2N2, Canada; umyang48@myumanitoba.ca (C.Y.); Zhongwei.Zou@umanitoba.ca (Z.Z.)

**Keywords:** *Brassica napus*, *Leptosphaeria maculans*, gene-for-gene interaction, temperature, *BONZAI1 (BON1)*, pathogenesis-related protein (PR)

## Abstract

Temperature is considered one of the crucial environmental elements in plant pathological interactions, and previous studies have indicated that there is a relationship between temperature change and host–pathogen interactions. The objective of this research is to investigate the link between temperature and the incompatible interactions of the host and pathogen. In this study, two *Leptosphaeria maculans* isolates (HCRT75 8-1 and HCRT77 7-2) and two *Brassica napus* genotypes (Surpass400 and 01-23-2-1) were selected. The selected *B. napus* genotypes displayed intermediate and resistant phenotypes. The inoculated seedlings were tested under three temperature conditions: 16 °C/10 °C, 22 °C/16 °C and 28 °C/22 °C (day/night: 16 h/8 h). Lesion measurements demonstrated that the necrotic lesions from the 28 °C/22 °C treatment were enlarged compared with the other two temperature treatments (i.e., 16 °C/10 °C and 22 °C/16 °C). The results of expression analysis indicated that the three temperature treatments displayed distinct differences in two marker genes (*PATHOGENESIS–RELATED (PR) 1* and *2*) for plant defense and one temperature-sensitive gene *BONZAI 1* (*BON1*). Additionally, seven dpi at 22 °C/16 °C appeared to be the optimal pre-condition for the induction of *PR1* and *2*. These findings suggest that *B. napus* responds to temperature changes when infected with *L. maculans*.

## 1. Introduction

Plants develop sets of mechanisms to combat the threat from phytopathogens. Plants secrete a set of metabolites, proteins and gene factors after the triggering of defense responses. According to two studies on the *Arabidopsis thaliana–Pseudomonas syringae* pv. tomato DC3000, the elicitation of innate immunity induces salicylic acid (SA), jasmonic acid (JA) and ethylene (ET) responsive genes [1,2]; *Brassica napus* was also found to express hormone/ROS-related signals when coping with the fungal pathogen *Leptosphaeria maculans* [3,4,5]. 

Plant disease epidemics are affected by various environmental factors, including the temperature, humidity and wind, and these factors can be crucial elements influencing the development of disease in nature. Among these factors, temperature is an important element which influences both hosts and pathogens. Moreover, there is molecular evidence suggesting that these organisms (i.e., hosts and pathogens) have developed various adaptive genetic backgrounds to interact with changes in temperature by evolution; as such, certain genes related to infection/defense can be regulated to change the general physiology of an organism when temperatures reach specific levels. 

In nature, there is plenty of evidence indicating that a change in temperature alters disease epidemics by changing the infectivity (from pathogens) and defense (from hosts). For example, the rust pathogen *Puccinia striiformis* is less effective when the temperature increases (starting at 15.4 °C), and the pathogen is unable to infect seedlings of wheat when the temperature is over 21 °C. Shayka et al., (2015) [6], by studying potato late blight, suggested that an oscillation of temperature as low as 5 °C was able to increase the infection efficiency, lesion growth development and sporulation. Iglesias et al., (2010) [7], found the maximal spore concentration of *Phytophthora infestans* within a temperature range of from 16 to 23 °C in Spain, and the optimal temperature for oomycete formation was 21 °C. In the *Brassica napus*–*Leptosphaeria maculans* pathosystem, an alteration in the temperature could also change the interaction between the host and fungus. One study based on the Effector–Triggered Immunity (ETI) between *Arabidopsis thaliana* and *Pseudomonas syringae* revealed that the hypersensitive response (HR) was suppressed at an elevated temperature of 28–30 °C compared with the ambient temperature (21–24 °C) [8]. Based on the *B. napus–L. maculans* pathosystem, Huang et al. (2006) [9], suggested that the fungus could grow faster and become more aggressive with an increased incubation temperature, and this happened in the case of both compatible and incompatible interactions. Studies have shown the connection between temperature and the expression of plant defense signaling pathways. A study regarding tobacco resistance against Tobacco Mosaic Virus (TMV) in 1992 [10] suggested that, at an elevated temperature (32 °C), tobacco compromised its salicylic acid (SA)-related resistance with a reduction in the *PR1* gene expression. Compared with a lower temperature (22 °C), both free SA and conjugated SA were increased and *PR1* expression was induced, which was able to cause necrotic lesions [10]. In *Arabidopsis*, the gene *BONZAI1* (*BON1*) has been found to modulate the plant defense in a temperature-sensitive manner. The *BON1* gene is part of the *COPINE* gene family, which supports plant growth and development, and negatively regulates plant defense and programmed cell death (PCD) [11,12,13,14]. The family of *BON* genes consists of three homologs: *BON1*, *2* and *3*. *BON1* plays a major role while the other two *BON* genes are more redundant. Moreover, the triple mutant of all three *BON* genes (*bon1bon2bon3*) of *Arabidopsis* also displays difficulty in germination [14]. Studies have suggested that *BON1* positively regulates plant growth at lower temperatures, and the mutation of *BON* genes induces excessive cell death at 22 °C [14,15,16]. *BON1* also represses an *R* gene named *SNC1* (*suppressor of NPR1, constitutive 1*), and the *BON1* mutant *bon1–1* induces constitutive resistant responses [17,18]. In the field, the temperature may change in each growing season and the strength of host resistance may be affected because of changes in the climate [19]. By considering all of the information mentioned above, it is possible to conduct a set of experiments to detect the effects of temperature on the defense of *B. napus* against the blackleg pathogen. 

In this study, **a set of experiments were conducted to explore the effects of temperature upon HR resistance against *L. maculans* races on *B. napus* genotypes at the seedling stage**. By analyzing the inoculated *B. napus* seedlings at three different growing temperatures, distinct patterns of fungal development were observed and molecular evidence suggested that the intrinsic signaling also responded differently at those temperatures.

## 2. Results

**Figure 1:**
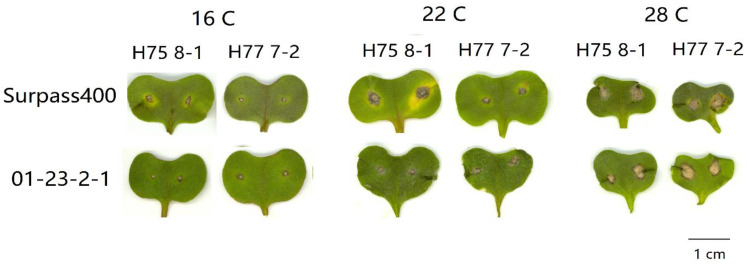
Lesion development from six pairs of *Brassica napus* cotyledon–*Leptosphaeria maculans* isolate inoculation: Surpass400–HCRT75 8-1/HCRT77 7-2 and 01-23-2-1–HCRT75 8-1/HCRT 77 7-2 at 11 days post-inoculation (dpi) with three temperature treatments: 16 °C/10 °C, 22 °C/16 °C, and 28 °C/22 °C (day/night: 16 h/8 h). Bar = 1cm.

**Figure 2:**
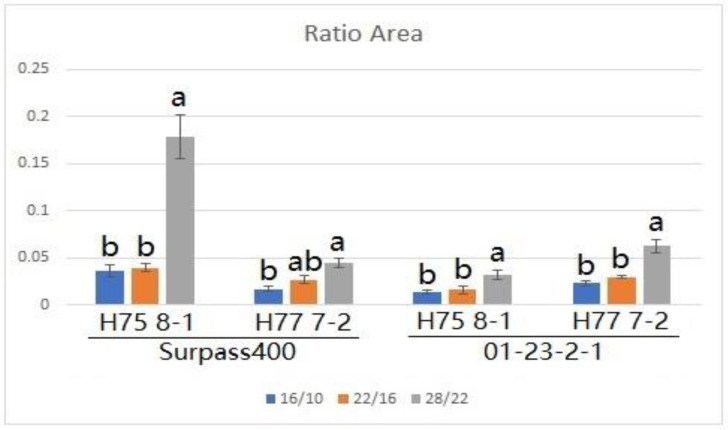
The extent of blackleg lesion development at 11 dpi from Surpass400 and 01-23-2-1 inoculated by the isolates HCRT75 8-1 and HCRT77 7-2. The inoculated plants were exposed to three temperature treatments: 16 °C/10 °C, 22 °C/16 °C and 28 °C/22 °C (day/night: 16 h/8 h). The lesion development was calculated by the ratio between the area of the lesion and the area of the cotyledon. Error bars represent standard error of the mean. Different lowercase letters suggest the significant differences among mean values (Fisher’s Least Significant Difference; *p* < 0.05). The results are based on three replicates in three independent experiments.

By measuring the lesions on the cotyledons after three temperature treatments, both Surpass400–H75 8-1/H77 7-2 and 01-23-2-1–H75 8-1/H77 7-2 exhibited phenotypes of incompatible interactions; as such, brownish necrotic lesions formed around the sites of inoculation (Figure 1). The *L. maculans* isolate HCRT75 8-1 (Genotype: avrLm1, AvrLm2, avrLm3, avrLm4, AvrLmJ1-5, AvrLm7, AvrLm6, avrLm9, AvrLm11, avrLepR1 and AvrLepR2) induced incompatible interaction upon Surpass400 (BLMR1/LepR3 and BLMR2/LepR2) by AvrLepR2-BLMR2/LepR2 interaction, and upon 01-23-2-1 (Rlm7) by AvrLm4-7–Rlm7 interaction, respectively. The *L. maculans* isolate HCRT77 7-2 (Genotype: AvrLm1, avrLm2, avrLm3, AvrLm4, AvrLmJ1-5, AvrLm7, AvrLm6, avrLm9, AvrLm11, avrLepR1 and avrLepR2) induced incompatible interaction upon Surpass400 (BLMR1/LepR3 and BLMR2/LepR2) by AvrLm1–BLMR1/LepR3 and upon 01-23-2-1 (Rlm7) by AvrLm4-7–Rlm7 interaction, respectively [20,21,22]. Generally, the results indicated that a higher temperature caused larger lesion sizes and, at 28 °C/22 °C, the lesions were the largest compared with the other two temperature treatments (Figure 2). Surpass400–H75 8-1, as the inoculation combination showing an intermediate phenotype (22 °C/16 °C), displayed an apparent increase in lesion development when responding to an increasing temperature. The other three cases, which were usually identified as resistant interactions (i.e., Surpass400–H77 7-2 and 01-23-2-1–H75 8-1/H77 7-2), displayed relatively mild increases in lesion development as the temperature became higher.

**Figure 3:**
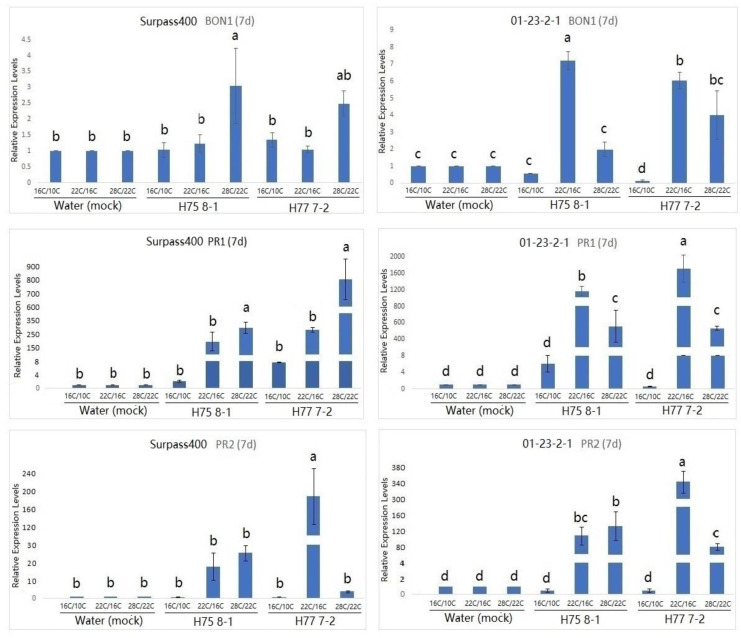
Gene expression of temperature-dependent regulator *BONZAI1 (BON1)*, *PATHOGENESIS-RELATED* (*PR)1* and *PATHOGENESIS-RELATED* (*PR)2* (in *B. napus*) in the regulation of hormonal signals at 7 dpi from Surpass400 and 01-23-2-1 inoculated by the blackleg isolates HCRT75 8-1 and HCRT77 7-2. The inoculated plants were exposed to three temperature treatments: 16 °C/10 °C, 22 °C/16 °C and 28 °C/22 °C (day/night: 16 h/8 h). The levels of the bars are the expression levels obtained from the inoculated cotyledons (inoculated by H75 8-1 and H77 7-2) compared to the cotyledons inoculated with water (assuming that the expression of each studied gene in the cotyledons inoculated with water is 1). Error bars represent standard error of the mean. Different lowercase letters suggest the significant differences among mean values (Fisher’s Least Significant Difference; *p* < 0.05). The results are based on three replicates in three independent experiments.

**Figure 4:**
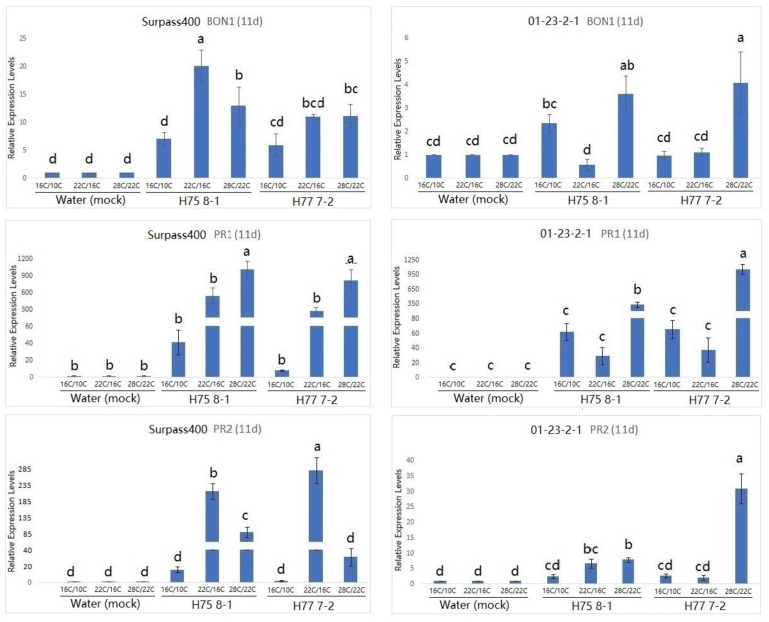
Gene expression of *BONZAI1 (BON1)*, *PATHOGENESIS-RELATED* (*PR)1* and *PATHOGENESIS-RELATED* (*PR)2* (in *B. napus*) in the regulation of hormonal signals at 11 dpi from Surpass400 and 01-23-2-1 inoculated by the blackleg isolates HCRT75 8-1 and HCRT77 7-2. The inoculated plants were exposed to three temperature treatments: 16 °C/10 °C, 22 °C/16 °C and 28 °C/22 °C (day/night: 16 h/8 h). The levels of the bars are the expression levels obtained from the inoculated cotyledons (inoculated by H75 8-1 and H77 7-2) compared to the cotyledons inoculated with water (assuming that the expression of each studied gene in the cotyledons inoculated with water is 1). Error bars represent standard error of the mean. Different lowercase letters suggest the significant differences among mean values (Fisher’s Least Significant Difference; *p* < 0.05). The results are based on three replicates in three independent experiments.

Studies have suggested that *BON* genes are related to the growth/defense balance at a low temperature (22 °C), and BON1 has been found to play a dominant role in repressing the plant defense [11,15]. As can be seen by the expression of *BON1* in Figure 3, *BON1* was more pronounced at the 22 °C/16 °C condition compared with the other two conditions at 7 dpi. Similar to at 7 dpi, *BON1* was still induced at 11 dpi when the plant was treated with the 28 °C/22 °C condition (Figure 4). Moreover, Surpass400–H75 8-1/H77 7-2 maintained a pronounced up-regulation in all three conditions at 11 dpi, while 01-23-2-1–H75 8-1 appeared to have higher levels of *BON1* at 16 °C/10 °C than at 22 °C/16 °C. Additionally, the gene also exhibited a high level of induction at 28 °C/22 °C.

According to previous studies on *Arabidopsis*, the up-regulation of the *PR1* gene was observed in the *bon1* mutants at the lower temperature (22 °C), suggesting that this gene might have the function of defense suppression in some conditions, such as temperature [23]. Looking at the *PR1* expression in Figure 3 and Figure 4, the genotype 01-23-2-1 exhibited the strongest induction at 7 dpi when the temperature was 22 °C/16 °C. On the other hand, for Surpass400, the optimal circumstance for the highest induction of *PR1* appeared to be at 11 dpi, at 28 °C/22 °C. It seemed that *PR1* expression in 01-23-2-1 synchronized with the expression of *BON1*, in which the trends of up- and down-regulation between these two genes were similar (both 7 and 11 dpi). On the other hand, *PR1* and *BON1* generally exhibited an antagonistic relationship in Surpass400, which matched with previous studies in *Arabidopsis*.

Another *PR* gene tested was *PR2*. *PR2* encodes an enzyme called beta-1, 3 glucanase 2. It is SA-responsive and functions as a regulator of sugar metabolism and fungal cell wall degradation [23,24,25]. According to Figure 3 and Figure 4, thermal fluctuation did not seem to have apparent effects on *PR2* expression. Except for Surpass400–H77 7-2 (at 7 dpi) and 01-23-2-1 – H77 7-2 (11 dpi), *PR2* was highly induced at 22 °C/16 °C, suggesting that, unlike *PR1*, *PR2* expression was only induced at 22 °C/16 °C. It seemed that *PR2* had no apparent connection with *BON1,* but it had generally lower expression in the 28 °C/22 °C condition, which indicated that *PR2* expression might be affected by other factors besides temperature and/or *BON1* regulation.

## 3. Discussion

Our experiments suggested that the HR lesions were increased at the higher temperature treatment (28 °C/22 °C). The expression profiles of *BON1*, *PR1* and *PR2* were distinct among three temperature conditions. The temperature 22 °C/16 °C is generally optimal for *PR1/2* to express. The *PR1/2* was also induced (higher than water inoculation) when the temperature treatment reached 28 °C/22 °C.

Previous evidence has suggested that a change in temperature is able to alter the general defense in plants. Malamy et al., (1992) [10], observed a reduction in the SA level and *PR1* expression in tobacco leaves (inoculated with tobacco mosaic virus (TMV)) when the temperature was increased to 32 °C from 22 °C; on the other hand, a higher temperature (i.e., 32 °C) enabled the virus to replicate and infect the host. Other signaling regulators, such as *BON* family genes, are involved in temperature-dependent regulation of plant defense.

According to previous studies on *Arabidopsis*, *BONZAI1* (*BON1*) is expressed at a lower temperature (22 °C) to regulate the general plant defense [14,15]. *BON* genes appear to regulate SA-related signals, such as *PR1*, and the expression of *BON1* represses SA-related pathogenesis-related proteins (*PR1*, *PR2*, and *PR5*) [12,15]. The results from lesion measurement matched those presented in previous studies of pathogenic development, with an increasing temperature-promoting pathogenic development and suppressing host defense [10,19,26,27]. The lesion size, especially that obtained from the Surpas400–H75 8-1 case, increased significantly at 28 °C/22 °C compared with 16 °C/10 °C and 22 °C/16 °C, whilst the other three cases (Surpass400–H77 7-2 and 01-23-2-1–H75 8-1/H77 7-2) also exhibited moderate increases in the lesion size. The enlargement of the lesion size on those genotypes suggested that the effect of the hypersensitive response (HR) on the suppression of fungal growth was mitigated when the temperature was raised. Previous studies have indicated that the temperature has effects on the strength of Effector–Triggered Immunity (ETI).* SNC1* (*suppressor of NPR1, constitutive 1*), as an *R* gene in *Arabidopsis*, is repressed by *BON1* on its promoter region [18]. *SNC1* has been found to play a role in defense against *Nicotiana benthamiana*, together with the *N* gene (another *R* gene specific for *N. benthamiana*). Moreover, the nuclear accumulation of *SNC1* and *N* genes is reduced when the temperature is elevated [28]. *SCN1* is also suppressed by factors other than *BON1* at a higher temperature. In *Arabidopsis*, HOPZ-ETI-DEFICIENT 1 (ZED1) and ZED1-related kinases (ZRKs) suppress *SNC1* expression at a temperature of 25 °C, and the mutation of *ZED1* was shown to activate defense genes *PR1* and *PR2* at 25 °C [29].

According to the qPCR results, the genes *BON1*, *PR1* and *PR2* were found to react to the thermal changes by observing the transcriptional analyses; however, some changes did not follow the indicated rules set out in previous studies. One remarkable discrepancy is that both *BON1* and *PR1* displayed a high level of induction at 28 °C/22 °C. Conversely, in previous research, the expression of these genes was shown to be lower than in the other low-temperature conditions.

In the 22 °C/16 °C condition, an antagonistic relationship between *BON1* and *PR1* appeared to be shown. *PR1* displayed a very high expression when the expression of *BON1* was not very high, it was more obvious from Surpass400 (except for 11 dpi, Surpass400 H75 8-1), and this finding matched that of the repression of *BON1* upon defense genes and cell death [11,12,15]. In the same condition (22 °C/16 °C, Surpass400), *PR1* and *2* were induced at 7 dpi and repressed in 11 dpi, which synchronized with the lower expression of *BON1* at 7 dpi and its high expression at 11 dpi.

Surprisingly, in the 28 °C/22 °C condition, both *BON1* and *PR1* were induced at 11 dpi. By considering the larger lesion size in this condition for both genotypes, the high expression of *PR1* can be explained as the physiological response towards more severe infectious situations, similar trends also happened to 01-23-2-1 at 22 °C/16 °C. The infected hosts with compatible interactions were found to have a high level of induction of defense-related genes at a later stage of infection compared with cases of incompatible interactions [3,30]. On the other hand, the induction of *BON1* was also observed, which did not display its negative regulation upon *PR1*, as mentioned in previous studies. It is possible that, in a high-temperature growth condition, the resistant *B. napus* genotypes obtain a homeostatic status, where both the activation and repression of defense mechanisms occur.

There are still many questions that need to be answered in order to explain the relationship between the regulation of plant defense and changing of temperature. This signaling system may be more complicated and depends on different species. In this article, *PR2* did not follow the presumed pattern of expression when changing the temperature, and other factors may affect the expression of downstream proteins like PR proteins. One possible explanation for this unusual situation is the homeostatic regulation between plant growth and defense. A plant body may suppress excessive defensive activities when there are few pathogens present inside. Plants may develop certain mechanisms, such as a guard model, to activate their resistance when a large amount of pathogen inoculum are recognized, since *R* gene-related defense is destructive to plant bodies [31]. In addition, ZED1 and ZRKs repress SNC1-triggered defense when there is no pathogen present. Moreover, these genes negatively regulate the SNC1-activated autoimmunity at an elevated temperature (relative to ambient temperature) [29].

## 4. Materials and Methods

### 4.1. Plant Cultivation and Temperature Treatments

Two *Brassica napus* genotypes (Surpass400 (*BLMR1/LepR3* and *BLMR2/RlmS*) and 01-23-2-1 (*Rlm7*)) were grown under three temperature conditions: 16 °C/10 °C (day/night: 16 h/8 h); 22 °C/16 °C (day/night: 16 h/8 h); 28 °C/22 °C (day/night: 16 h/8 h). All flats with seedlings were grown under 22 °C/16 °C (day/night: 16 h/8 h) first, and the seedlings to be tested under the other two conditions were moved into the growth cabinets 24 h before inoculation.

### 4.2. Pathogen Inoculation

Two *L. maculans* isolates were selected for inoculation: HCRT75 8-1 (Genotype: avrLm1, AvrLm2, avrLm3, avrLm4, AvrLmJ1, AvrLm7, AvrLm6, avrLm9, AvrLm11, avrLepR1 and AvrLepR2) and HCRT77 7-2 (Genotype: AvrLm1, avrLm2, avrLm3, AvrLm4, AvrLmJ1, AvrLm7, AvrLm6, avrLm9, AvrLm11, avrLepR1 and avrLepR2).

The cotyledons of *B. napus* cultivars were inoculated seven days after sowing (cotyledon stage) by puncture inoculation. Each lobe of cotyledons was punctured by a sterile needle twice from each side, to have four inoculation points on each seedling of the canola plant.

### 4.3. Lesion Measurement

The cotyledons at 11 days post-inoculation (dpi) were scanned and the lesion size was measured by ImageJ (National Institutes of Health, Bethesda, MD, USA).

### 4.4. Gene Expression Analysis

Frozen cotyledons (7 and 11 dpi) were ground in liquid nitrogen with pestles and mortars. The total RNA was extracted with TRI reagent (Sigma-Aldrich) (St. Louis, MO USA). According to the manual, the total RNA was purified by DNaseI treatment with a recombinant DNaseI, RNase-free kit (Roche). Purified RNA was used to synthesize cDNA by employing the GOScript Reverse Transcription System (Promega). The cDNA stock solution was diluted to 100 ng/µL. Quantitative-PCR was performed by loading 1 µL of cDNA (100 ng) into the 10 µL reaction system of the IQTM SYBR^®^ Green Supermix (BioRad, Hercules, CA, USA). The experiments were based on three biological replicates. The RT-qPCR experiments were run by Touch Real-Time PCR System (BioRad, Hercules, CA, USA).

The qPCR program used for all of the analysed genes (except for *COI1* and *ACO1*) was 95 °C for 3 min, followed by 39 cycles of 95 °C for 15 s and 60 °C for 20 s. This was followed by a melting curve analysis.

All qPCR primers are compiled in Supplementary Table S1. The relative level of gene expression was analysed with the 2-ΔΔCT method described by Livak and Schmittgen, (2001) [32]. Actin was used as a reference gene to normalize the expression of the target genes.

### 4.5. Statistical Analysis

Unless specified, the analyses of the samples used at least three biological replicates. The statistical analyses were performed using the Tukey ANOVA method with SAS 9.4 software. 

## 5. Conclusions

Taken together, the evidence displayed in this article suggests that infected genotypes growing at a higher temperature cause **larger lesion sizes on the resistant genotype by shaping the effects of the hypersensitive response.** Expression analysis revealed that, in a higher temperature condition (28 °C/22 °C), both ***BON1* and *PR1*** were **triggered at 7 and 11 dpi**, and were presumed to have an antagonistic relationship with each other based on previous studies on *Arabidopsis*. The results indicated that, at a higher temperature, *B. napus* seems to display a balance between the plant defense and growth mechanism, at the same time as exhibiting incompatible interactions, from which the expression of defense-repressing factor *BON1* and defense gene *PR1* coincides during a pathogen attack.

## Data Availability

The original contributions presented in the study are included in the article/supplementary materials, further inquiries can be directed to the corresponding author/s.

## Supplementary Materials

The following are available online at https://www.mdpi.com/article/10.3390/plants10050843/s1, Table S1: Primer list used for quantitative-PCR.
